# Enhancement of Antitumor Vaccination by Targeting Dendritic Cell-Related IL-10

**DOI:** 10.3389/fimmu.2018.01923

**Published:** 2018-09-03

**Authors:** Diana Llopiz, Marta Ruiz, Leyre Silva, Pablo Sarobe

**Affiliations:** ^1^Programa de Inmunología e Inmunoterapia, Centro de Investigación Médica Aplicada (CIMA), Universidad de Navarra, Pamplona, Spain; ^2^Instituto de Investigación Sanitaria de Navarra (IdiSNA), Pamplona, Spain

**Keywords:** antitumor therapeutic vaccination, dendritic cells, interleukin 10, immunosuppressive cells, PD-L1, type I IFN

## Abstract

Understanding mechanisms associated to dendritic cell (DC) functions has allowed developing new antitumor therapeutic vaccination strategies. However, these vaccines have demonstrated limited clinical results. Although the low immunogenicity of tumor antigens used and the presence of tumor-associated suppressive factors may in part account for these results, intrinsic vaccine-related factors may also be involved. Vaccines modulate DC functions by inducing activating and inhibitory signals that determine ensuing T cell responses. In this mini review, we focus on IL-10, inhibitory cytokine induced in DC upon vaccination, which defines a suppressive cell subset, discussing its implications as a potential target in combined vaccination immunotherapies.

## Dendritic cells in therapeutic vaccination

Since William Coley treated cancer patients with bacterial extracts to activate immunity, therapeutic vaccination has been considered a promising immunotherapeutic approach (1). During the last decades, we have witnessed the identification of dendritic cells (DC) as professional antigen presenting cells (2), characterization of their biological properties (3) and subsets (4), as well as the development of new techniques and tools to directly purify them (5) or differentiate from peripheral blood precursors (6). Therefore, a plethora of vaccination clinical trials has been carried out, either through *in vivo* administration of antigens and adjuvants, or *ex vivo*-prepared antigen-loaded DC (7, 8). Advances related to understanding those receptors and biological pathways involved in antigen capture and DC activation have allowed developing new vaccines, in terms of improving antigen targeting (9) or vaccine formulation (10), as well as improving direct DC isolation or differentiation from precursors, antigen loading and maturation (11). Despite these efforts in improving vaccine immunogenicity, those strategies reaching clinical phases, have provided limited clinical results (7). Accordingly, there is only a single licensed therapeutic cancer vaccine, Provenge, approved for castration resistant prostate cancer (12).

Characterization of the tumor microenvironment has clearly demonstrated the presence of immunosuppressive mechanisms which render T-cells dysfunctional (13, 14), partially accounting for vaccine failure. However, there are vaccine intrinsic factors which have not been fully elucidated and whose characterization may also explain in part these results. A variety of protocols have been used in vaccination clinical trials, with differences in parameters such as the type of antigens, its loading method in case of DC vaccines, the adjuvant or maturation protocol and the type/stage of disease and patients vaccinated, among others (15). This heterogeneity has made difficult to draw solid conclusions to identify those factors linking properties of vaccines with the ensuing immunological and clinical results (16). Thus, although characterization of the vaccine product is usually a requisite for its release, commonly analyzed parameters, usually related to pro-immunogenic vaccine properties, have not completely revealed the relevant clues on vaccine immunogenicity (17).

Activation of naive T-cells by DC requires antigen recognition on MHC molecules, co-stimulatory signals and polarizing cytokines, according to the three-signal model (18). Although immature, resting DC usually lack these molecules, in the case of infections, contact with microbial pathogens leads to antigen capture and simultaneous sensing of pathogen associated molecular patterns (PAMPs), inducing thus the upregulation of genes involved in eliciting immunogenic responses (3). Similarly, in the tumor setting, danger associated molecular patterns (DAMPs) and other signals released by dying tumor cells are known to promote DC activation (19, 20). However, as in many biological processes, recognition of these signals by DC may also lead to upregulation of genes associated with negative feedback mechanisms, regulating thus immune activation. These include expression of membrane-bound co-inhibitory ligands (21, 22) that modulate signal 2, together with the release of soluble molecules (cytokines and metabolites) (23, 24) modulating signal 3. Vaccines rely either on the administration of antigens and immunostimulatory molecules (adjuvants) which will reach DC *in vivo* (25), or on administering DC that have been antigen-loaded and stimulated *ex vivo* (15). Similarly to infectious processes, DC may upregulate these control elements upon vaccine administration or during the DC preparation process (in the case of DC vaccines). Therefore, understanding these feedback mechanisms and delineation of optimized strategies to block them may allow developing more immunogenic vaccines. In this Minireview we will focus on IL-10, a cytokine regulating many functions, describing those mechanisms that control their induction on DC, its effect on these cells during vaccination as well as the rationale to best block their inhibitory effect with therapeutic vaccination purposes.

## IL-10: an inhibitory molecule in vaccination

Among cytokines reported to down-regulate the activation of antitumor immune responses, IL-10 plays a prominent role. IL-10 is a pleiotropic cytokine traditionally considered as immunosuppressive for antigen presenting cell functions and concomitant priming of T-cells (26). Although initially considered a cytokine produced by Th2 cells (27) or Tregs, it is now known that it is produced not only by other lymphocytic subsets, but also by cells of innate immunity, including DC and macrophages (28). Stimuli such as Toll-like receptor (TLR) ligands or CD40 ligand (CD40L), usually present in microbial pathogens or induced because of inflammation, have been included as adjuvants in different vaccination strategies (25). Although they have a high capacity to promote DC maturation and release pro-immunogenic cytokines like IL-12 (29), they may also induce IL-10, even with synergistic effects in some cases (30–32). There is an inverse relationship between IL-10 and IL-12 production by DC, which has been attributed to different mechanisms, including MAP kinase activation (33–35) and transcription factors NFIL3 (36) and DC-SCRIPT (37). Interestingly, DC-activating adjuvants have different cytokine-producing profiles, which may vary depending on the cytokine considered. Therefore, not all ligands have the same capacity to induce IL-10 (38), depending on the receptor involved and its associated signaling pathway. Indeed, although there are differences between murine and human studies because of the type of DC subsets and the corresponding TLR repertoire (39), some stimuli like TLR molecules (TLR2, TLR4, TLR7, or TLR9, among others) strongly induce IL-10 production (32, 40, 41). However, others like TLR3 ligand poly(I:C) or CD40 agonists (CD40L or antiCD40 antibodies) are considered poorer IL-10 inducers, mainly when used alone (42, 43). DC receptors responsible for sensing microbial or endogenous inflammatory/danger signals can be grouped according to the mediator molecules and the corresponding signaling pathways used to induce DC activation (44). Most TLR ligands signal through MyD88, with the exception of TLR3, which relies on TRIF for signal transduction, and TLR4, which depends on MyD88- and TRIF-dependent pathways (45). Other non-TLR DC receptors, such as lectins receptors, Nod-like receptors or RIG-like receptors, use other sets of signaling molecules, including Syk, ASC and MAVS (46). Thus, signaling through MyD88 or Syk vs. signaling through TRIF (47) may explain the distinct capacity to produce IL-10 by different ligands. Furthermore, some of these pathways lead to the activation of different MAP kinases (p38, Jun, ERK, among others) that promote or inhibit IL-10 and IL-12 production, depending on the relative activation balance between kinases. In this respect, signaling pathways resulting in the activation of ERK (33–35) and MK2 (48) have been shown to induce IL-10. In addition, the receptor and signaling pathway not only determine IL-10 production, but also the susceptibility of DC to autocrine effects mediated by its own IL-10. It has been demonstrated that IL-10 induced in DC after stimulation by ligands that signal through the MyD88 pathway (e.g., TLR4 or TLR9 ligands), inhibits DC functions, such as the secretion of cytokines IL-6 and IL-1β and expression of IL-12 p35, IL-23 p19, TNF-α, and IFN-β mRNA (49). In contrast, IL-10 induced by curdlan (a Dectin-1 ligand) does not affect DC functions. Finally, IL-10 receptor expression may also represent an additional control mechanism. Surface expression of IL-10 receptor is downregulated upon LPS-induced maturation (50), potentially explaining the increased susceptibility of immature vs. mature DC to IL-10 inhibitory effects. All these results suggest that there is an intricate network of interactions involving pathways regulating IL-10 production as well as those implicated in the susceptibility to its effects, which has to be considered when analyzing the role of IL-10 during DC activation.

IL-10 has been described in many cancer patients as a poor prognostic factor (51–54). It can be detected in serum (52, 53) and in the tumor (51), produced by tumor cells (55, 56) as well as by infiltrating hematopoietic cells, including myeloid (57) and lymphocytic subsets (58–61). Although IL-10 has an inhibitory impact for antigen presenting cells, contradictory effects have been reported in the case of antitumor T-cells (62–64). In this regard, it has been demonstrated that IL-10 increases functional properties of already activated effector CD8 T-cells. In fact, in different murine tumor models, including mammary carcinoma, skin squamous carcinoma, and several transplantable models, such as breast, colon, and melanoma tumors, administration of IL-10 inhibits tumor growth, promoting antitumor functions of effector T-cells, increasing tumor infiltration, IFN-gamma production and lytic molecules (65, 66). However, regarding priming of naive T-cells during initial stages, IL-10 has been considered detrimental. This is due to its inhibitory role on antigen presenting cells at different levels, such as migration (67), expression of co-stimulatory molecules (68), production of Th1 polarizing cytokines (57, 69) and blocking cross-priming and priming with low-affinity peptides of a self/tumor-antigen and concomitant T-cell activation (70). Therefore, to consider IL-10 as a target in tumor immunotherapy, the immunological context has to be taken into account. In those settings where already primed effector cells exist, IL-10 may potentiate their properties (65, 66). However, if T-cell priming or generation of an antitumor immune response is needed, as in vaccination, therapeutic benefit would be achieved by IL-10 blockade. Indeed, preclinical data regarding IL-10 blockade has demonstrated an enhancement of vaccine immunogenicity, not only in the cancer setting (43, 71, 72) but also in other models (41, 73, 74). Although, as previously mentioned, IL-10 can be expressed by different cell subsets, we and others (43, 75) have demonstrated that antigen presenting cell-derived IL-10 down-regulates the elicitation of Th1 responses. Indeed, vaccine-dependent induction of IFN-gamma-producing T-cells is greatly enhanced by IL-10 blockade when using adjuvants promoting IL-10 production by DC (75), which in the case of tumors results in a stronger therapeutic effect (43). Thus, although different IL-10 sources coexist in the tumor setting, DC-derived IL-10 seems to determine vaccination efficacy.

## IL-10 as a marker of suppressive DC

**Figure 1:**
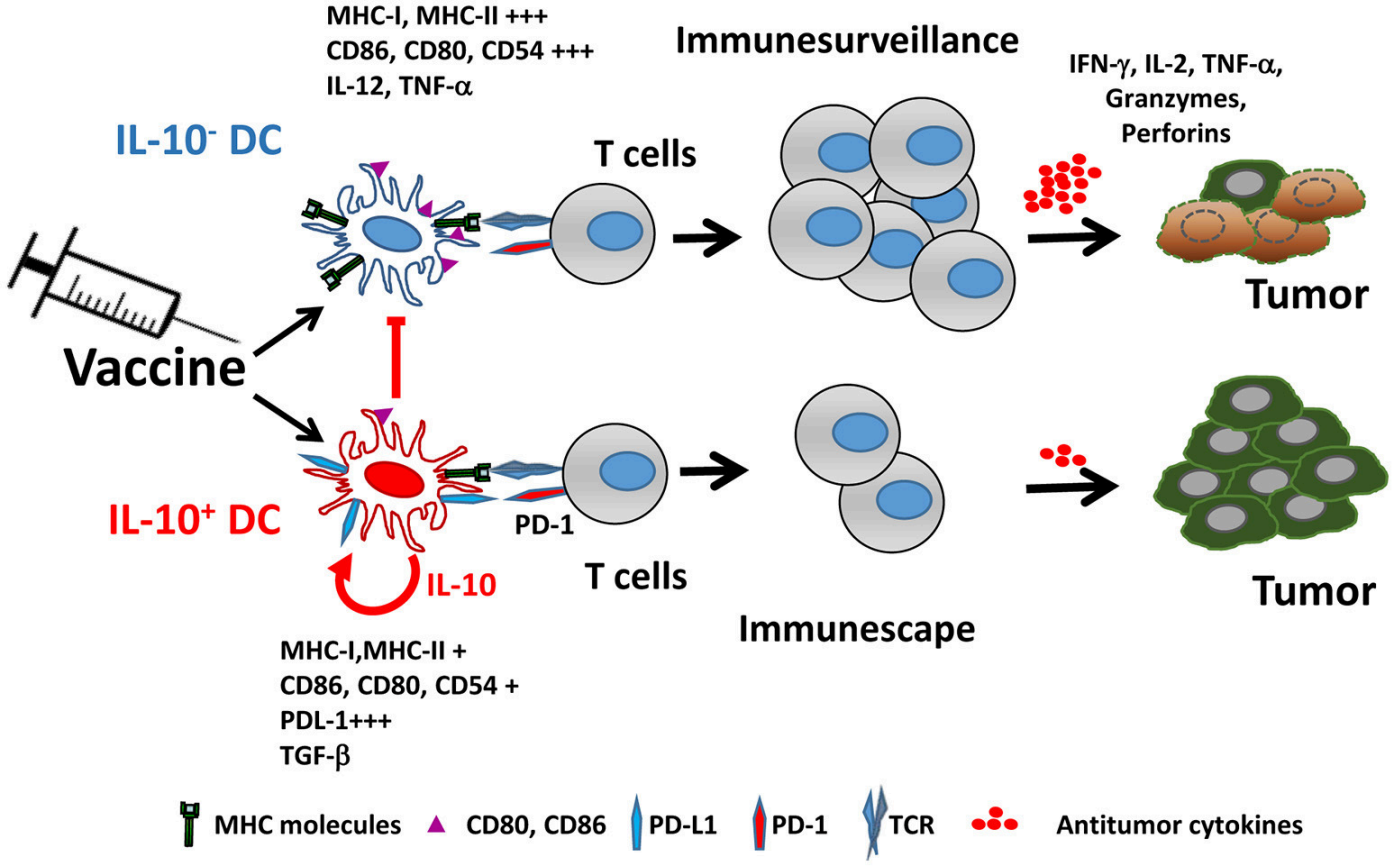
Vaccine-induced IL-10 production defines two different dendritic cell populations. Adjuvants included in vaccines promote the generation of two DC populations identified by their capacity to produce IL-10. IL-10^+^ DC are characterized by poorer expression of MHC and co-stimulatory molecules, upregulation of PD-L1 and immunosuppressive cytokines, resulting in impaired ability to activate antitumor T cells, as opposed to IL-10^−^ DC, which retain full capacity to trigger functional antitumor T cell responses. Moreover, IL-10^+^ DC inhibit antigen-presenting functions of IL-10^−^ DC.

IL-10 production by DC not only affects their functional properties, but also identifies a subpopulation characterized by many immunosuppressive features, both at the phenotypical and functional levels. Mice vaccinated with IL-10-inducing adjuvants have an IL-10-producing DC subset (from now on IL-10+ DC), which is almost absent in unvaccinated mice. IL-10+ DC are characterized by poorer expression of co-stimulatory molecules and inflammatory cytokines, as well as by upregulation of co-inhibitory ligands such as PD-L1, resulting thus in a cell population with much lower T-cell stimulatory capability (76) (Figure 1). Interestingly, in addition to their low antigen presenting capacity, IL-10+ DC also suppress antigen presentation by third-party cells, reinforcing their inhibitory role. Some of these features of IL-10+ DC have been also observed in persistent viral infection models (77), suggesting that generation of this DC subset may not be specific of vaccines and operates in other settings.

There are no mechanistic experiments in the vaccination setting explaining the presence of different DC populations according to their IL-10 production. Indeed, it is not known whether the vaccine generates both types of DC from different precursors or the same DC subset may behave differently. However, in the viral model it has been demonstrated that, whereas IL-10- DC are originated from conventional DC precursors, the source of IL-10+ DC are monocytes differentiated as a consequence of inflammatory mediators, which are subsequently endowed with suppressive properties by type I IFN (78). This association between IL-10 production by DC and the presence of type I IFN, is in agreement with the vaccine setting, where many adjuvants known to induce IL-10 (ligands of TLR4, TLR7, and TLR9) also induce type I IFN (79), a cytokine that determines IL-10 levels (80). Moreover, IL-10+ DC have higher ISG expression, indicating a stronger response to type I IFN. IL-10 and type I IFN production have been linked as regulatory mechanisms, and blockade of IFN signal leads to a diminished IL-10 expression (81, 82). However, there may be additional factors behind IL-10 induction, since potent type I IFN inducers, such as the TLR3 ligand poly(I:C), are poor at inducing IL-10. Similarly, expression of other inhibitory molecules, such as PD-L1, also depends on type I IFN, in agreement with the pivotal role of this cytokine in generating the immunosuppressive effect on this DC subpopulation (81, 82). Interestingly, in addition to these immunoregulatory effects, it has been reported that type I IFN is required by DC to induce rejection of immunogenic tumors, supporting the induction of adaptive responses (83). Moreover, efficacy of therapeutic strategies such as vaccines (84) or other approaches relying on cross-presentation of tumor antigens by DC (85) also depends on type I IFN. Overall, these results suggest that type I IFN plays a dual role on DC-mediated tumor immunity, by promoting the generation of antitumor responses, but at the same time inducing regulatory mechanisms -including IL-10- to balance the magnitude of these responses (86).

In addition to the murine setting, there are several examples of suppressive DC in human studies, having in common the production of IL-10. Besides those protocols aimed at preparing tolerogenic DC by incubation with IL-10 in the therapy of autoimmune diseases, which are not the scope of this Minireview, there are also inflammatory conditions where induction of IL-10 takes place and results in similar suppressive functions. As an example, *in vitro* differentiation of monocytes in an inflammatory environment, such as the presence of TLR ligands (e.g., LPS or R848), led to generation of tolerogenic CD14+ DC expressing high IL-10 and PD-L1 levels (87) in a STAT3-dependent manner. Moreover, combined DC stimulation with potent TLR ligands results in partial inhibition, due to IL-10 induced by some of these compounds (42). More interestingly, the relevance of vaccine-derived IL-10 has been also demonstrated in the clinical setting. A recent paper (88) analyzing properties of monocyte-derived DC used as vaccines in prostate cancer patients reported that IL-10, in addition to CD14, and MCP-1 and MDC chemokines, identified a gene signature that could discriminate between patients responding or not to the vaccine. Authors found that clinical and strong immunological responses correlated with low expression of molecules belonging to this gene signature, some of them, like CD14 and IL-10, already described as tolerogenic markers of DC. In addition to monocyte-derived DC, vaccines based on DC directly isolated from blood have been also used. When analyzing the cell composition of these vaccines, Bakdash et al. found a BDCA1+ DC population positive for CD14, which is elevated in patients as compared with healthy donors (89). These cells are a DC subset that, although overlaps in many factors with monocytes and classical BDCA1+ DC, can be considered as a distinct population, characterized by displaying suppressive properties. Therefore, they have been suggested as responsible for hampering vaccine efficacy in patients. Interestingly, besides CD14 expression [as in DC previously mentioned (88)], these DC are characterized by stronger IL-10 secretion than monocytes or classical BDCA1+ DC upon LPS stimulation, reagent also used to induce DC maturation in (88). Also in the clinical setting, we reported results from a vaccination pilot clinical trial of patients suffering from chronic hepatitis C by using a DC vaccine (90). Poor Th1 immune responses were detected in vaccinated patients. Although disease status could have played a role in these results, we detected higher levels of CD14+ DC and a stronger IL-10 production by the DC vaccine prepared from patients, as opposed to DC obtained from healthy individuals, in agreement with aforementioned clinical studies. Interestingly, blockade of IL-10 during vaccine preparation restored *in vitro* production of Th1 responses in these patients, suggesting again a relevant role of IL-10 in vaccine efficacy.

Therefore, considering the relevance of IL-10 during therapeutic vaccination, different blockade strategies have been used, depending on the type of vaccine. As summarized in Table 1, for those vaccines relying on direct antigen administration, antibodies blocking IL-10 or IL-10R have been used in most cases. When using DC administration, in addition to direct IL-10/IL-10R blockade, genetic ablation of IL-10/IL-10R expression and pharmacological inhibition of pathways leading to IL-10 production have been also used. Despite these different options in preclinical studies, only antibodies against IL-10, in combination with a TLR9 ligand, have advanced to clinical phases for the treatment of patients with advanced malignancies.

**Table 1:** Strategies aimed at blocking IL-10 for combination with antitumor vaccines.

**IL-10 blocking strategy**	**Vaccine**	**Tumor**	**Reference**
**PRECLINICAL**			
Anti-IL-10 antibodies	Plasmid encoding MIP3α-hgp100	B16F10	(91)
	DC pulsed with gp100 peptide	B16F10	(92)
	DC pulsed with tumor cells	MC38	(93)
Anti-IL-10R antibodies	OVA + Imiquimod	B16-OVA	(43)
	HPV E7 peptide + LPS	TC-1	(72)
Soluble IL-10R	DC pulsed with tumor cells	B16F10	(94)
siRNA targeting IL-10	DC pulsed with E7 peptide	TC-1	(95)
siRNA targeting IL-10R	DC pulsed with E7 peptide	TC-1	(96)
	DC pulsed with MART-1 peptide	Human melanoma (*in vitro*)	(97)
shRNA targeting IL-10	DC pulsed with tumor cells	MC38	(98)
Aptamer targeting IL-10R	Monotherapy (no vaccine)	CT26	(99)
Inhibitors of IL-10-inducing pathways: p38 MAPK inhibitor	DC pulsed with tumor cells	B16F10	(100)
COX2 inhibitor	DC pulsed with tumor cells	B16F10	(100)
Retinoic acid receptor alpha inhibitor	DC pulsed with tumor cells	B16F10	(101)
**CLINICAL**			
Anti-IL-10 antibodies	TLR9 ligand	Advanced tumors	clinicaltrials.gov NCT02731742

In addition to IL-10 as a target, description of this IL-10+ DC subset and its immunosuppressive properties allows the identification of other relevant molecular mechanisms involved in their inhibitory effect. Therefore, these molecules could be potentially amenable to modulation as a strategy to enhance vaccine potency. Among them, PD-L1 is an interesting upregulated target, since there are already approved therapies directed at this pathway (102). By using samples from patients with different types of tumors (hepatocellular carcinoma and glioblastoma, among others), it has been demonstrated that PD-L1 expression is regulated by IL-10 (103, 104). However, in our hands in vaccination experiments in murine models, combination of vaccine with IL-10 blockade did not modify PD-L1 expression on DC, despite a decrease in the percentage of IL-10+ DC (76), pointing at PD-L1 as an independent target and providing an additional opportunity for DC modulation. Accordingly, combined blockade of IL-10 and PD1/PD-L1 clearly potentiated vaccine immunogenicity, resulting in a greater therapeutic antitumor effect (76, 84). These results are in agreement with equivalent experiments carried out in viral models *in vitro* (105) and *in vivo* (106). In addition to these two important suppressive pathways, other inhibitory molecules, including enzymes (IDO) (77), cytokines (TGF-beta), ligands for receptors found on T-cells (HVEM) or inhibitory intracellular molecules (IRAK-3) (78), have been described in this IL-10+ DC subset. Co-expression of these negative factors has been already reported in other examples of DC with poor stimulatory capacity (107, 108), indicating that they are commonly operating in settings where T-cell responses are not fully activated, and suggesting that combined blockade of these mechanisms may improve DC functions, with special relevance in vaccination protocols.

## Conclusion and future directions

Different immunosuppressive elements present at the tumor microenvironment have been described, demonstrating that they may hamper effector functions of tumor-infiltrating lymphocytes. These mechanisms would partially account for the limited effect of therapeutic vaccines, suggesting that combination therapies that include vaccines plus blockade of these elements may increase their efficacy. However, together with these elements, additional inhibitory pathways induced by the vaccine are triggered. Many of them, such as IL-10 production, exert their effects at the level of DC, by impairing their antigen presenting functions and negatively regulating T-cell activation. Some of the already identified vaccine-induced suppressive elements present in IL-10+ DC, are common to those operating at the tumor level, allowing the design of new combinatorial vaccination strategies based on drugs currently approved or in development. Therefore, future vaccination strategies, besides highly immunogenic and properly formulated and adjuvanted antigens, should incorporate blockade of IL-10 and additional inhibitory elements, enhancing thus vaccine potency and associated therapeutic efficacy.

## Author contributions

DL and PS conceived, wrote the manuscript and did the figures. MR and LS contributed to manuscript editing and final revision.

### Conflict of interest statement

The authors declare that the research was conducted in the absence of any commercial or financial relationships that could be construed as a potential conflict of interest.

## References

[1] Pardoll DM Cancer vaccines. Nat Med. (1998) 4(5 Suppl):525–31. 10.1038/nm0598supp-525 9585204

[2] Steinman RM. The dendritic cell system and its role in immunogenicity. Annu Rev Immunol. (1991) 9:271–96. 10.1146/annurev.iy.09.040191.001415 1910679

[3] Banchereau J Briere F Caux C Davoust J Lebecque S Liu YJ. Immunobiology of dendritic cells. Annu Rev Immunol. (2000) 18:767–811. 10.1146/annurev.immunol.18.1.767 10837075

[4] Guilliams M Dutertre CA Scott CL McGovern N Sichien D Chakarov S. Unsupervised high-dimensional analysis aligns dendritic cells across tissues and species. Immunity (2016) 45:669–84. 10.1016/j.immuni.2016.08.015 27637149 PMC5040826

[5] Dzionek A Fuchs A Schmidt P Cremer S Zysk M Miltenyi S. BDCA-2, BDCA-3, and BDCA-4: three markers for distinct subsets of dendritic cells in human peripheral blood. J Immunol. (2000) 165:6037–46. 10.4049/jimmunol.165.11.6037 11086035

[6] Sallusto F Lanzavecchia A. Efficient presentation of soluble antigen by cultured human dendritic cells is maintained by granulocyte/macrophage colony-stimulating factor plus interleukin 4 and downregulated by tumor necrosis factor alpha. J Exp Med. (1994) 179:1109–18. 10.1084/jem.179.4.1109 8145033 PMC2191432

[7] Melero I Gaudernack G Gerritsen W Huber C Parmiani G Scholl S. Therapeutic vaccines for cancer: an overview of clinical trials. Nat Rev Clin Oncol. (2014) 11:509–24. 10.1038/nrclinonc.2014.111 25001465

[8] Butterfield LH. Cancer vaccines. BMJ (2015) 350:h988. 10.1136/bmj.h988 25904595 PMC4707521

[9] Cohn L Delamarre L. Dendritic cell-targeted vaccines. Front Immunol. (2014) 5:255. 10.3389/fimmu.2014.00255 24910635 PMC4039009

[10] Moyer TJ Zmolek AC Irvine DJ. Beyond antigens and adjuvants: formulating future vaccines. J Clin Invest. (2016) 126:799–808. 10.1172/JCI81083 26928033 PMC4767337

[11] Palucka K Banchereau J. Dendritic-cell-based therapeutic cancer vaccines. Immunity (2013) 39:38–48. 10.1016/j.immuni.2013.07.004 23890062 PMC3788678

[12] Kantoff PW Higano CS Shore ND Berger ER Small EJ Penson DF. Sipuleucel-T immunotherapy for castration-resistant prostate cancer. N Engl J Med. (2010) 363:411–22. 10.1056/NEJMoa1001294 20818862

[13] Kerkar SP Restifo NP. Cellular constituents of immune escape within the tumor microenvironment. Cancer Res. (2012) 72:3125–30. 10.1158/0008-5472.CAN-11-4094 22721837 PMC6327310

[14] Crespo J Sun H Welling TH Tian Z Zou W. T cell anergy, exhaustion, senescence, and stemness in the tumor microenvironment. Curr Opin Immunol. (2013) 25:214–21. 10.1016/j.coi.2012.12.003 23298609 PMC3636159

[15] Palucka K Banchereau J. Cancer immunotherapy via dendritic cells. Nat Rev Cancer (2012) 12:265–77. 10.1038/nrc3258 22437871 PMC3433802

[16] Santos PM Butterfield LH. Dendritic cell-based cancer vaccines. J Immunol. (2018) 200:443–9. 10.4049/jimmunol.1701024 29311386 PMC5880540

[17] Bol KF Schreibelt G Gerritsen WR de Vries IJ Figdor CG. Dendritic cell-based immunotherapy: state of the art and beyond. Clin Cancer Res. (2016) 22:1897–906. 10.1158/1078-0432.CCR-15-1399 27084743

[18] Kapsenberg ML. Dendritic-cell control of pathogen-driven T-cell polarization. Nat Rev Immunol. (2003) 3:984–93. 10.1038/nri1246 14647480

[19] Apetoh L Ghiringhelli F Tesniere A Obeid M Ortiz C Criollo A. Toll-like receptor 4-dependent contribution of the immune system to anticancer chemotherapy and radiotherapy. Nat Med. (2007) 13:1050–9. 10.1038/nm1622 17704786

[20] Woo SR Fuertes MB Corrales L Spranger S Furdyna MJ Leung MY. STING-dependent cytosolic DNA sensing mediates innate immune recognition of immunogenic tumors. Immunity (2014) 41:830–42. 10.1016/j.immuni.2014.10.017 25517615 PMC4384884

[21] Groschel S Piggott KD Vaglio A Ma-Krupa W Singh K Goronzy JJ. TLR-mediated induction of negative regulatory ligands on dendritic cells. J Mol Med. (2008) 86:443–55. 10.1007/s00109-008-0310-x 18253710 PMC2556182

[22] Yao S Jiang L Moser EK Jewett LB Wright J Du J. Control of pathogenic effector T-cell activities in situ by PD-L1 expression on respiratory inflammatory dendritic cells during respiratory syncytial virus infection. Mucosal Immunol. (2015) 8:746–59. 10.1038/mi.2014.106 25465101 PMC4632244

[23] Grohmann U Fallarino F Puccetti P. Tolerance, DCs and tryptophan: much ado about IDO. Trends Immunol. (2003) 24:242–8. 10.1016/S1471-4906(03)00072-3 12738417

[24] Braun D Longman RS Albert ML. A two-step induction of indoleamine 2,3 dioxygenase (IDO) activity during dendritic-cell maturation. Blood (2005) 106:2375–81. 10.1182/blood-2005-03-0979 15947091 PMC1895261

[25] Dowling JK Mansell A. Toll-like receptors: the swiss army knife of immunity and vaccine development. Clin Transl Immunol. (2016) 5:e85. 10.1038/cti.2016.22 27350884 PMC4910119

[26] Moore KW de Waal Malefyt R Coffman RL O'Garra A. Interleukin-10 and the interleukin-10 receptor. Annu Rev Immunol. (2001) 19:683–765. 10.1146/annurev.immunol.19.1.683 11244051

[27] Fiorentino DF Bond MW Mosmann TR. Two types of mouse T helper cell. IV. Th2 clones secrete a factor that inhibits cytokine production by Th1 clones. J Exp Med. (1989) 170:2081–95. 10.1084/jem.170.6.2081 2531194 PMC2189521

[28] Saraiva M O'Garra A. The regulation of IL-10 production by immune cells. Nat Rev Immunol. (2010) 10:170–81. 10.1038/nri2711 20154735

[29] Napolitani G Rinaldi A Bertoni F Sallusto F Lanzavecchia A. Selected Toll-like receptor agonist combinations synergistically trigger a T helper type 1-polarizing program in dendritic cells. Nat Immunol. (2005) 6:769–76. 10.1038/ni1223 15995707 PMC3760217

[30] Boonstra A Rajsbaum R Holman M Marques R Asselin-Paturel C Pereira JP. Macrophages and myeloid dendritic cells, but not plasmacytoid dendritic cells, produce IL-10 in response to MyD88- and TRIF-dependent TLR signals, and TLR-independent signals. J Immunol. (2006) 177:7551–8. 10.4049/jimmunol.177.11.7551 17114424

[31] Mitchell D Yong M Schroder W Black M Tirrell M Olive C. Dual stimulation of MyD88-dependent Toll-like receptors induces synergistically enhanced production of inflammatory cytokines in murine bone marrow-derived dendritic cells. J Infect Dis. (2010) 202:318–29. 10.1086/653499 20524851

[32] Hirata N Yanagawa Y Ebihara T Seya T Uematsu S Akira S. Selective synergy in anti-inflammatory cytokine production upon cooperated signaling via TLR4 and TLR2 in murine conventional dendritic cells. Mol Immunol. (2008) 45:2734–42. 10.1016/j.molimm.2008.02.010 18372043

[33] Agrawal S Agrawal A Doughty B Gerwitz A Blenis J Van Dyke T. Cutting edge: different Toll-like receptor agonists instruct dendritic cells to induce distinct Th responses via differential modulation of extracellular signal-regulated kinase-mitogen-activated protein kinase and c-Fos. J Immunol. (2003) 171:4984–9. 10.4049/jimmunol.171.10.4984 14607893

[34] Dillon S Agrawal S Banerjee K Letterio J Denning TL Oswald-Richter K. Yeast zymosan, a stimulus for TLR2 and dectin-1, induces regulatory antigen-presenting cells and immunological tolerance. J Clin Invest. (2006) 116:916–28. 10.1172/JCI27203 16543948 PMC1401484

[35] Qian C Jiang X An H Yu Y Guo Z Liu S. TLR agonists promote ERK-mediated preferential IL-10 production of regulatory dendritic cells (diffDCs), leading to NK-cell activation. Blood (2006) 108:2307–15. 10.1182/blood-2006-03-005595 16778140

[36] Smith AM Qualls JE O'Brien K Balouzian L Johnson PF Schultz-Cherry S. A distal enhancer in Il12b is the target of transcriptional repression by the STAT3 pathway and requires the basic leucine zipper (B-ZIP) protein NFIL3. J Biol Chem. (2011) 286:23582–90. 10.1074/jbc.M111.249235 21566115 PMC3123121

[37] Sondergaard JN van Heeringen SJ Looman MWG Tang C Triantis V Louche P. Dendritic cells actively limit interleukin-10 production under inflammatory conditions via DC-SCRIPT and dual-specificity phosphatase 4. Front Immunol. (2018) 9:1420. 10.3389/fimmu.2018.01420 29988341 PMC6023963

[38] Samarasinghe R Tailor P Tamura T Kaisho T Akira S Ozato K. Induction of an anti-inflammatory cytokine, IL-10, in dendritic cells after toll-like receptor signaling. J Interferon Cytokine Res. (2006) 26:893–900. 10.1089/jir.2006.26.893 17238832

[39] Iwasaki A Medzhitov R. Toll-like receptor control of the adaptive immune responses. Nat Immunol. (2004) 5:987–95. 10.1038/ni1112 15454922

[40] Jang S Uematsu S Akira S Salgame P. IL-6 and IL-10 induction from dendritic cells in response to *Mycobacterium tuberculosis* is predominantly dependent on TLR2-mediated recognition. J Immunol. (2004) 173:3392–7. 10.4049/jimmunol.173.5.3392 15322203

[41] Castro AG Neighbors M Hurst SD Zonin F Silva RA Murphy E. Anti-interleukin 10 receptor monoclonal antibody is an adjuvant for T helper cell type 1 responses to soluble antigen only in the presence of lipopolysaccharide. J Exp Med. (2000) 192:1529–34. 10.1084/jem.192.10.1529 11085755 PMC2193194

[42] Bogunovic D Manches O Godefroy E Yewdall A Gallois A Salazar AM. TLR4 engagement during TLR3-induced proinflammatory signaling in dendritic cells promotes IL-10-mediated suppression of antitumor immunity. Cancer Res. (2011) 71:5467–76. 10.1158/0008-5472.CAN-10-3988 21730023 PMC3156386

[43] Llopiz D Aranda F Diaz-Valdes N Ruiz M Infante S Belsue V. Vaccine-induced but not tumor-derived Interleukin-10 dictates the efficacy of Interleukin-10 blockade in therapeutic vaccination. Oncoimmunology (2015) 5:e1075113. 10.1080/2162402X.2015.1075113 27057445 PMC4801433

[44] Brubaker SW Bonham KS Zanoni I Kagan JC. Innate immune pattern recognition: a cell biological perspective. Annu Rev Immunol. (2015) 33:257–90. 10.1146/annurev-immunol-032414-112240 25581309 PMC5146691

[45] Akira S Uematsu S Takeuchi O. Pathogen recognition and innate immunity. Cell (2006) 124:783–801. 10.1016/j.cell.2006.02.015 16497588

[46] Kawai T Akira S. The roles of TLRs, RLRs and NLRs in pathogen recognition. Int Immunol. (2009) 21:317–37. 10.1093/intimm/dxp017 19246554 PMC2721684

[47] Slack EC Robinson MJ Hernanz-Falcon P Brown GD Williams DL Schweighoffer E. Syk-dependent ERK activation regulates IL-2 and IL-10 production by DC stimulated with zymosan. Eur J Immunol. (2007) 37:1600–12. 10.1002/eji.200636830 17458858

[48] Soukup K Halfmann A Le Bras M Sahin E Vittori S Poyer F. The MAPK-activated kinase MK2 attenuates dendritic cell-mediated Th1 differentiation and autoimmune encephalomyelitis. J Immunol. (2015) 195:541–52. 10.4049/jimmunol.1401663 26078274

[49] Chang J Kunkel SL Chang CH. Negative regulation of MyD88-dependent signaling by IL-10 in dendritic cells. Proc Natl Acad Sci USA. (2009) 106:18327–32. 10.1073/pnas.0905815106 19815506 PMC2775313

[50] Corinti S Albanesi C la Sala A Pastore S Girolomoni G. Regulatory activity of autocrine IL-10 on dendritic cell functions. J Immunol. (2001) 166:4312–8. 10.4049/jimmunol.166.7.4312 11254683

[51] Fujieda S Lee K Sunaga H Tsuzuki H Ikawa H Fan GK. Staining of interleukin-10 predicts clinical outcome in patients with nasopharyngeal carcinoma. Cancer (1999) 85:1439–45. 10193932

[52] Bohlen H Kessler M Sextro M Diehl V Tesch H. Poor clinical outcome of patients with Hodgkin's disease and elevated interleukin-10 serum levels. Clinical significance of interleukin-10 serum levels for Hodgkin's disease. Ann Hematol. (2000) 79:110–3. 10.1007/s002770050564 10803931

[53] Chau GY Wu CW Lui WY Chang TJ Kao HL Wu LH Serum interleukin-10 but not interleukin-6 is related to clinical outcome in patients with resectable hepatocellular carcinoma. Ann Surg. (2000) 231:552–8. 10.1097/00000658-200004000-00015 10749617 PMC1421032

[54] Lech-Maranda E Baseggio L Bienvenu J Charlot C Berger F Rigal D. Interleukin-10 gene promoter polymorphisms influence the clinical outcome of diffuse large B-cell lymphoma. Blood (2004) 103:3529–34. 10.1182/blood-2003-06-1850 14701701

[55] Gastl GA Abrams JS Nanus DM Oosterkamp R Silver J Liu F. Interleukin-10 production by human carcinoma cell lines and its relationship to interleukin-6 expression. Int J Cancer (1993) 55:96–101. 10.1002/ijc.2910550118 8344757

[56] Kruger-Krasagakes S Krasagakis K Garbe C Schmitt E Huls C Blankenstein T. Expression of interleukin 10 in human melanoma. Br J Cancer (1994) 70:1182–5. 10.1038/bjc.1994.469 7981073 PMC2033698

[57] Ruffell B Chang-Strachan D Chan V Rosenbusch A Ho CM Pryer N. Macrophage IL-10 blocks CD8+ T cell-dependent responses to chemotherapy by suppressing IL-12 expression in intratumoral dendritic cells. Cancer Cell (2014) 26:623–37. 10.1016/j.ccell.2014.09.006 25446896 PMC4254570

[58] Kryczek I Liu R Wang G Wu K Shu X Szeliga W. FOXP3 defines regulatory T cells in human tumor and autoimmune disease. Cancer Res. (2009) 69:3995–4000. 10.1158/0008-5472.CAN-08-3804 19383912

[59] Wakkach A Augier S Breittmayer JP Blin-Wakkach C Carle GF. Characterization of IL-10-secreting T cells derived from regulatory CD4+CD25+ cells by the TIRC7 surface marker. J Immunol. (2008) 180:6054–63. 10.4049/jimmunol.180.9.6054 18424726

[60] Ouyang FZ Wu RQ Wei Y Liu RX Yang D Xiao X. Dendritic cell-elicited B-cell activation fosters immune privilege via IL-10 signals in hepatocellular carcinoma. Nat Commun. (2016) 7:13453. 10.1038/ncomms13453 27853178 PMC5118541

[61] Shalapour S Lin XJ Bastian IN Brain J Burt AD Aksenov AA. Inflammation-induced IgA+ cells dismantle anti-liver cancer immunity. Nature (2017) 551:340–5. 10.1038/nature24302 29144460 PMC5884449

[62] Groux H Bigler M de Vries JE Roncarolo MG. Inhibitory and stimulatory effects of IL-10 on human CD8+ T cells. J Immunol. (1998) 160:3188–93. 9531274

[63] Mocellin S Panelli MC Wang E Nagorsen D Marincola FM. The dual role of IL-10. Trends Immunol. (2003) 24:36–43. 10.1016/S1471-4906(02)00009-1 12495723

[64] Geginat J Larghi P Paroni M Nizzoli G Penatti A Pagani M. The light and the dark sides of Interleukin-10 in immune-mediated diseases and cancer. Cytokine Growth Factor Rev. (2016) 30:87–93. 10.1016/j.cytogfr.2016.02.003 26980675

[65] Mumm JB Emmerich J Zhang X Chan I Wu L Mauze S. IL-10 elicits IFNgamma-dependent tumor immune surveillance. Cancer Cell (2011) 20:781–96. 10.1016/j.ccr.2011.11.003 22172723

[66] Emmerich J Mumm JB Chan IH LaFace D Truong H McClanahan T. IL-10 directly activates and expands tumor-resident CD8(+) T cells without de novo infiltration from secondary lymphoid organs. Cancer Res. (2012) 72:3570–81. 10.1158/0008-5472.CAN-12-0721 22581824

[67] Demangel C Bertolino P Britton WJ. Autocrine IL-10 impairs dendritic cell (DC)-derived immune responses to mycobacterial infection by suppressing DC trafficking to draining lymph nodes and local IL-12 production. Eur J Immunol. (2002) 32:994–1002. 10.1002/1521-4141(200204)32:4<994:AID-IMMU994>3.0.CO;2-6 11920565

[68] Ding L Linsley PS Huang LY Germain RN Shevach EM. IL-10 inhibits macrophage costimulatory activity by selectively inhibiting the up-regulation of B7 expression. J Immunol. (1993) 151:1224–34. 7687627

[69] D'Andrea A Aste-Amezaga M Valiante NM Ma X Kubin M Trinchieri G. Interleukin 10 (IL-10) inhibits human lymphocyte interferon gamma-production by suppressing natural killer cell stimulatory factor/IL-12 synthesis in accessory cells. J Exp Med. (1993) 178:1041–8. 10.1084/jem.178.3.1041 8102388 PMC2191152

[70] Nizzoli G Larghi P Paroni M Crosti MC Moro M Neddermann P. IL-10 promotes homeostatic proliferation of human CD8(+) memory T cells and, when produced by CD1c(+) DCs, shapes naive CD8(+) T-cell priming. Eur J Immunol. (2016) 46:1622–32. 10.1002/eji.201546136 27129615

[71] Lu H Wagner WM Gad E Yang Y Duan H Amon LM. Treatment failure of a TLR-7 agonist occurs due to self-regulation of acute inflammation and can be overcome by IL-10 blockade. J Immunol. (2010) 184:5360–7. 10.4049/jimmunol.0902997 20308630

[72] Chen S Wang X Wu X Wei MQ Zhang B Liu X. IL-10 signalling blockade at the time of immunization inhibits Human papillomavirus 16 E7 transformed TC-1 tumour cells growth in mice. Cell Immunol. (2014) 290:145–51. 10.1016/j.cellimm.2014.06.002 24983823

[73] Brooks DG Lee AM Elsaesser H McGavern DB Oldstone MB. IL-10 blockade facilitates DNA vaccine-induced T cell responses and enhances clearance of persistent virus infection. J Exp Med. (2008) 205:533–41. 10.1084/jem.20071948 18332180 PMC2275377

[74] Bai F Town T Qian F Wang P Kamanaka M Connolly TM. IL-10 signaling blockade controls murine West Nile virus infection. PLoS Pathog. (2009) 5:e1000610. 10.1371/journal.ppat.1000610 19816558 PMC2749443

[75] Darrah PA Hegde ST Patel DT Lindsay RW Chen L Roederer M. IL-10 production differentially influences the magnitude, quality, and protective capacity of Th1 responses depending on the vaccine platform. J Exp Med. (2010) 207:1421–33. 10.1084/jem.20092532 20530206 PMC2901071

[76] Llopiz D Ruiz M Infante S Villanueva L Silva L Hervas-Stubbs S. IL-10 expression defines an immunosuppressive dendritic cell population induced by antitumor therapeutic vaccination. Oncotarget (2017) 8:2659–71. 10.18632/oncotarget.13736 27926522 PMC5356831

[77] Wilson EB Kidani Y Elsaesser H Barnard J Raff L Karp CL. Emergence of distinct multiarmed immunoregulatory antigen-presenting cells during persistent viral infection. Cell Host Microbe (2012) 11:481–91. 10.1016/j.chom.2012.03.009 22607801 PMC3359873

[78] Cunningham CR Champhekar A Tullius MV Dillon BJ Zhen A de la Fuente JR. Type I, and Type II interferon coordinately regulate suppressive dendritic cell fate and function during viral persistence. PLoS Pathog (2016) 12:e1005356. 10.1371/journal.ppat.1005356 26808628 PMC4726812

[79] Noppert SJ Fitzgerald KA Hertzog PJ. The role of type I interferons in TLR responses. Immunol Cell Biol. (2007) 85:446–57. 10.1038/sj.icb.7100099 17667935

[80] Howes A Taubert C Blankley S Spink N Wu X Graham CM. Differential production of type I IFN determines the reciprocal levels of IL-10 and proinflammatory cytokines produced by C57BL/6 and BALB/c macrophages. J Immunol. (2016) 197:2838–53. 10.4049/jimmunol.1501923 27549173 PMC5026030

[81] Wilson EB Yamada DH Elsaesser H Herskovitz J Deng J Cheng G. Blockade of chronic type I interferon signaling to control persistent LCMV infection. Science (2013) 340:202–7. 10.1126/science.1235208 23580528 PMC3704950

[82] Teijaro JR Ng C Lee AM Sullivan BM Sheehan KC Welch M. Persistent LCMV infection is controlled by blockade of type I interferon signaling. Science (2013) 340:207–11. 10.1126/science.1235214 23580529 PMC3640797

[83] Diamond MS Kinder M Matsushita H Mashayekhi M Dunn GP Archambault JM. Type I interferon is selectively required by dendritic cells for immune rejection of tumors. J Exp Med. (2011) 208:1989–2003. 10.1084/jem.20101158 21930769 PMC3182061

[84] Villanueva L Silva L Llopiz D Ruiz M Iglesias T Lozano T. The Toll like receptor 4 ligand cold-inducible RNA-binding protein as vaccination platform against cancer. Oncoimmunology (2018) 7:e1409321. 10.1080/2162402X.2017.1409321 29632721 PMC5889285

[85] Schiavoni G Mattei F Gabriele L. Type I Interferons as stimulators of DC-mediated cross-priming: impact on anti-tumor response. Front Immunol. (2013) 4:483. 10.3389/fimmu.2013.00483 24400008 PMC3872318

[86] Snell LM McGaha TL Brooks DG. Type I interferon in chronic virus infection and cancer. Trends Immunol. (2017) 38:542–57. 10.1016/j.it.2017.05.005 28579323 PMC8059441

[87] Wolfle SJ Strebovsky J Bartz H Sahr A Arnold C Kaiser C. PD-L1 expression on tolerogenic APCs is controlled by STAT-3. Eur J Immunol. (2011) 41:413–24. 10.1002/eji.201040979 21268011

[88] Castiello L Sabatino M Ren J Terabe M Khuu H Wood LV. Expression of CD14, IL10, and tolerogenic signature in dendritic cells inversely correlate with clinical and immunologic response to TARP vaccination in prostate cancer patients. Clin Cancer Res. (2017) 23:3352–64. 10.1158/1078-0432.CCR-16-2199 28073842 PMC5496805

[89] Bakdash G Buschow SI Gorris MA Halilovic A Hato SV Skold AE. Expansion of a BDCA1+CD14+ myeloid cell population in melanoma patients may attenuate the efficacy of dendritic cell vaccines. Cancer Res. (2016) 76:4332–46. 10.1158/0008-5472.CAN-15-1695 27325645

[90] Zabaleta A D'Avola D Echeverria I Llopiz D Silva L Villanueva L. Clinical testing of a dendritic cell targeted therapeutic vaccine in patients with chronic hepatitis C virus infection. Mol Ther Methods Clin Dev. (2015) 2:15006. 10.1038/mtm.2015.6 26029717 PMC4444996

[91] Gordy JT Luo K Francica B Drake C Markham RB. Anti-IL-10-mediated enhancement of antitumor efficacy of a dendritic cell-targeting MIP3alpha-gp100 vaccine in the B16F10 mouse melanoma model is dependent on type I interferons. J Immunother. (2018) 41:181–9. 10.1097/CJI.0000000000000212 29334492 PMC5891382

[92] Kalli F Machiorlatti R Battaglia F Parodi A Conteduca G Ferrera F. Comparative analysis of cancer vaccine settings for the selection of an effective protocol in mice. J Transl Med. (2013) 11:120. 10.1186/1479-5876-11-120 23663506 PMC3659084

[93] Rossowska J Anger N Kicielinska J Pajtasz-Piasecka E Bielawska-Pohl A Wojas-Turek J. Temporary elimination of IL-10 enhanced the effectiveness of cyclophosphamide and BMDC-based therapy by decrease of the suppressor activity of MDSCs and activation of antitumour immune response. Immunobiology (2015) 220:389–98. 10.1016/j.imbio.2014.10.009 25454807

[94] Marchi LH Paschoalin T Travassos LR Rodrigues EG. Gene therapy with interleukin-10 receptor and interleukin-12 induces a protective interferon-gamma-dependent response against B16F10-Nex2 melanoma. Cancer Gene Ther. (2010) 18:110–22. 10.1038/cgt.2010.58 20885448

[95] Kim JH Kang TH Noh KH Bae HC Ahn YH Lee YH. Blocking the immunosuppressive axis with small interfering RNA targeting interleukin (IL)-10 receptor enhances dendritic cell-based vaccine potency. Clin Exp Immunol. (2011) 165:180–9. 10.1111/j.1365-2249.2011.04410.x 21592111 PMC3142643

[96] Ahn YH Hong SO Kim JH Noh KH Song KH Lee YH. The siRNA cocktail targeting interleukin 10 receptor and transforming growth factor-beta receptor on dendritic cells potentiates tumour antigen-specific CD8(+) T cell immunity. Clin Exp Immunol. (2015) 181:164–78. 10.1111/cei.12620 25753156 PMC4469167

[97] Chhabra A Chakraborty NG Mukherji B. Silencing of endogenous IL-10 in human dendritic cells leads to the generation of an improved CTL response against human melanoma associated antigenic epitope, MART-1 27-35. Clin Immunol. (2008) 126:251–9. 10.1016/j.clim.2007.11.011 18249038 PMC2352151

[98] Rossowska J Anger N Szczygiel A Mierzejewska J Pajtasz-Piasecka E. Reprogramming the murine colon cancer microenvironment using lentivectors encoding shRNA against IL-10 as a component of a potent DC-based chemoimmunotherapy. J Exp Clin Cancer Res. (2018) 37:126. 10.1186/s13046-018-0799-y 29954431 PMC6025815

[99] Berezhnoy A Stewart CA McNamara JO 2nd Thiel W Giangrande P Trinchieri G. Isolation and optimization of murine IL-10 receptor blocking oligonucleotide aptamers using high-throughput sequencing. Mol Ther. (2012) 20:1242–50. 10.1038/mt.2012.18 22434135 PMC3369303

[100] Jarnicki AG Conroy H Brereton C Donnelly G Toomey D Walsh K. Attenuating regulatory T cell induction by TLR agonists through inhibition of p38 MAPK signaling in dendritic cells enhances their efficacy as vaccine adjuvants and cancer immunotherapeutics. J Immunol. (2008) 180:3797–806. 10.4049/jimmunol.180.6.3797 18322186

[101] Galvin KC Dyck L Marshall NA Stefanska AM Walsh KP Moran B. Blocking retinoic acid receptor-alpha enhances the efficacy of a dendritic cell vaccine against tumours by suppressing the induction of regulatory T cells. Cancer Immunol Immunother. (2013) 62:1273–82. 10.1007/s00262-013-1432-8 23657628 PMC11029272

[102] Gong J Chehrazi-Raffle A Reddi S Salgia R. Development of PD-1 and PD-L1 inhibitors as a form of cancer immunotherapy: a comprehensive review of registration trials and future considerations. J Immunother Cancer (2018) 6:8. 10.1186/s40425-018-0316-z 29357948 PMC5778665

[103] Wu K Kryczek I Chen L Zou W Welling TH. Kupffer cell suppression of CD8+ T cells in human hepatocellular carcinoma is mediated by B7-H1/programmed death-1 interactions. Cancer Res. (2009) 69:8067–75. 10.1158/0008-5472.CAN-09-0901 19826049 PMC4397483

[104] Bloch O Crane CA Kaur R Safaee M Rutkowski MJ Parsa AT. Gliomas promote immunosuppression through induction of B7-H1 expression in tumor-associated macrophages. Clin Cancer Res. (2013) 19:3165–75. 10.1158/1078-0432.CCR-12-3314 23613317 PMC3742575

[105] Porichis F Hart MG Zupkosky J Barblu L Kwon DS McMullen A. Differential impact of PD-1 and/or interleukin-10 blockade on HIV-1-specific CD4 T cell and antigen-presenting cell functions. J Virol. (2014) 88:2508–18. 10.1128/JVI.02034-13 24352453 PMC3958087

[106] Brooks DG Ha SJ Elsaesser H Sharpe AH Freeman GJ Oldstone MB. IL-10 and PD-L1 operate through distinct pathways to suppress T-cell activity during persistent viral infection. Proc Natl Acad Sci USA. (2008) 105:20428–33. 10.1073/pnas.0811139106 19075244 PMC2629263

[107] Gordon JR Li F Nayyar A Xiang J Zhang X. CD8 alpha+, but not CD8 alpha-, dendritic cells tolerize Th2 responses via contact-dependent and -independent mechanisms, and reverse airway hyperresponsiveness, Th2, and eosinophil responses in a mouse model of asthma. J Immunol. (2005) 175:1516–22. 10.4049/jimmunol.175.3.1516 16034089

[108] Han Y Chen Z Yang Y Jiang Z Gu Y Liu Y. Human CD14+ CTLA-4+ regulatory dendritic cells suppress T-cell response by cytotoxic T-lymphocyte antigen-4-dependent IL-10 and indoleamine-2,3-dioxygenase production in hepatocellular carcinoma. Hepatology (2014) 59:567–79. 10.1002/hep.26694 23960017

